# Clinical Findings in Diabetes Mellitus Patients with COVID-19

**DOI:** 10.1155/2021/7830136

**Published:** 2021-01-08

**Authors:** Ting Guo, Qinxue Shen, Xiaoli Ouyang, Wei Guo, Jinhua Li, Wenlong He, Bo Yu, Chenfang Wu, Zhiguo Zhou, Hong Luo, Hong Peng

**Affiliations:** ^1^Department of Respiratory and Critical Care Medicine, The Second Xiangya Hospital of Central-South University, Changsha, Hunan 410011, China; ^2^Research Unit of Respiratory Disease, Central-South University, Changsha, Hunan 410011, China; ^3^The Respiratory Disease Diagnosis and Treatment Center of Hunan Province, Changsha, Hunan 410011, China; ^4^Department of Critical Care Medicine, The Second Xiangya Hospital of Central-South University, Changsha, Hunan 410011, China; ^5^Department of Respiratory and Critical Care Medicine, The First Hospital of Changsha, Changsha, Hunan 410000, China

## Abstract

**Backgrounds:**

Diabetes mellitus (DM) is one of the most common comorbidities in patients with coronavirus disease (COVID-19). We aim to summarize the clinical features of DM patients with COVID-19 and find out potential factors associated with severe disease.

**Methods:**

In this retrospective, single-center study, the medical records of patients with COVID-19 in Changsha, Hunan, China, from January 21, 2020, to February 19, 2020, were reviewed. Epidemiological information, clinical features, and outcomes were compared between DM patients admitted to the intensive care unit (ICU) or not.

**Results:**

A total of 241 patients confirmed with COVID-19 were enrolled, including 19 DM patients. There were more patients in DM group admitted to the ICU than non-DM group (36.8% vs. 15.8%, *P* = 0.045). Compared with non-DM group in the ICU, there were more female patients from DM group in the ICU (85.7% vs. 31.4%, *P* = 0.024). On admission, the mean level of glycated hemoglobin A1c (HbA1c) was higher in the ICU DM patients than that in the non-ICU DM patients (8.5% vs. 7.1%). There were more DM patients with proteinuria in the ICU group than the non-ICU group (57.1% vs. 33.3%). Twelve DM patients (63.2%) changed diabetic therapy during hospitalization, and all DM patients admitted to the ICU used insulin. As of March 14, all 19 DM patients have been discharged, and no death occurred.

**Conclusions:**

DM patients with COVID-19 are vulnerable to severe disease, especially for female patients. High levels of HbA1c and proteinuria could be potential risk factors for severe COVID-19 in DM patients. In addition to timely systemic therapy, the control of blood glucose and proper diabetic therapy is essential to improve the prognosis of severe DM patients with COVID-19.

## 1. Introduction

Severe acute respiratory syndrome coronavirus 2 (SARS-CoV-2) is a novel coronavirus that has typical features of the coronavirus family and is highly pathogenic to humans [1]. The large-scale transmission of SARS-CoV-2 since December 2019 from Wuhan, China, has resulted in over twenty million cases with infections and nine hundred thousand deaths worldwide [2]. World Health Organization (WHO) named the related disease as coronavirus disease 2019 (COVID-19) and assessed the COVID-19 outbreak as a global pandemic [3]. COVID-19 has drawn intensive attention and poses a threat to global public health. Diabetes mellitus (DM) is one of the most frequent comorbidities in patients with COVID-19 and is related to serious outcomes including intensive care unit (ICU) admission, invasive ventilation, and death [4, 5]. However, few clinical studies focusing on DM patients with COVID-19 have been reported, and associated factors with severe COVID-19 in DM patients were not clear yet.

In this retrospective study, we aimed to summarize and analyze the clinical features of DM patients with COVID-19 in Changsha, Hunan Province, China, from January 21, 2020, to February 19, 2020. We hope to find out potential factors associated with severe disease in DM patients with COVID-19.

## 2. Materials and Methods

### 2.1. Study Design and Participants

In this retrospective study, patients with laboratory-confirmed SARS-CoV-2 infection from January 21, 2020, to February 19, 2020, at the Public Health Clinic Center of Changsha, Hunan Province, China, according to WHO interim guidance were enrolled. The Public Health Clinic Center of Changsha is a hospital specializing in infectious disease. According to the arrangement of the Chinese government, COVID-19 patients were admitted centrally to the hospital from the whole of Changsha without selectivity. Clinical outcomes were followed up to March 14, 2020.

This study was approved by the Medical Ethical Committee of the Second Xiangya Hospital of Central-South University (Approved Number: KL-2020003) and Public Health Clinic Center of Changsha (Approved Number: KL-2020005), which waived the requirement for patients' informed consent referring to the CIOMS guideline.

Clinical information was collected from medical records. In this retrospective study, we divided patients with COVID-19 into the DM group and the non-DM group. Demographic information, clinical features, laboratory results, and imaging characteristics were compared. More importantly, we compared the differences between DM patients admitted to the ICU or not.

### 2.2. Definitions

Based on the New Coronavirus Pneumonia Prevention and Control Program (version six) from the National Health Commission of China, COVID-19 patients were divided into mild patients, moderate patients, severe patients, and critical patients. Mild COVID-19 was defined as mild clinical symptoms without imaging findings of pneumonia. Moderate COVID-19 was defined as fever and respiratory symptoms with imaging findings of pneumonia. Severe COVID-19 was defined as any one of the following criteria: (1) respiratory distress with a respiratory rate ≥ 30 breaths/min, (2) pulse oximeter oxygen saturation (SpO_2_) ≤ 93% in the resting state, and (3) PaO_2_/FiO_2_ ≤ 300 mmHg (1 mmHg = 0.133 kPa). And critical COVID-19 was defined as any one of the following criteria: (1) respiratory failure in need of mechanical ventilation, (2) shock, and (3) with other organ failures. Severe patients as well as critical patients were admitted to the ICU according to the guidelines from the National Health Commission of China.

The patients with a history of Wuhan exposure were defined as those with prior tourism or residence in Wuhan within 14 days before symptoms onset.

### 2.3. Statistical Analysis

Continuous variables were described as mean (ranges) and categorical variables as count (%). For laboratory results, we also assessed whether the measurements were outside the normal range. Means (SD) were compared with *t*-tests. Categorical variables were compared by the *χ*^2^ test or Fisher's exact test. All statistical analyses were performed using SPSS (version 26.0). *P* < 0.5 was considered statistically significant.

## 3. Results

### 3.1. Comparisons between COVID-19 Patients with DM and without DM

By Feb 19, 2020, 241 admitted hospital patients had confirmed COVID-19 infection in Changsha, 19 (7.9%) of them were DM patients, and 222 (92.1%) were non-DM patients. Baseline data for all the patients enrolled are shown in Table 1. Compared with non-DM patients, DM patients were older (62.1 years vs. 43.8 years, *P* < 0.0001). DM patients had more underlying diseases than non-DM patients (73.7% vs. 29.7%, *P* < 0.0001), especially about hypertension (63.2% vs. 10.8%, *P* < 0.0001) and coronary heart disease (CHD) (15.8% vs. 2.7%, *P* = 0.024). There were no differences of symptoms including cough, dyspnea, and fever on admission between the two groups. As for laboratory results on admission, DM patients had a higher percentage of proteinuria (42.1% vs. 5.9%, *P* < 0.0001) and a higher level of C-creative protein (CRP) (44.8 mg/L vs. 25.8 mg/L, *P* = 0.003). There were more patients in DM group admitted to the ICU than non-DM group (36.8% vs. 15.8%, *P* = 0.045). The female proportion in DM patients with ICU admission was 85.7%. While in the group of non-DM patients admitted to the ICU, the female proportion was 31.4%. There was a statistical significance between the two groups (*P* = 0.024). The mean time of symptoms onset to negative reverse transcription-polymerase chain reaction (RT-PCR) was 22.6 days (ranges 8-50 days) in DM group, and the time was shorter in non-DM patients (14.3 days, ranges 3-18 days) (*P* = 0.004). There was no difference of mortality in DM and non-DM groups.

### 3.2. Comparisons between COVID-19 DM Patients Admitted to the ICU or Not

#### 3.2.1. Demographic and Symptomatic Characteristics

A total of 19 DM patients with COVID-19 were included (mean age, 62.1 years [range, 42-76 years]; ten female). Ten patients had an exposure history of Wuhan before illness onset. Three patients were diagnosed with DM for the first time (patient 3, patient 7, and patient 18) (Supplementary material (available here)). Seven DM patients were admitted to the ICU (36.8%). Hypertension was the most frequent comorbidities (63.2%), and two patients had ever used angiotensin-converting enzyme inhibitors (ACEI) or angiotensin II type-I receptor blockers (ARBs) (patient 1 and patient 7). Two patients had chronic viral hepatitis B (patient 11 and patient 18), and one patient had liver cirrhosis (patient 14). Patient 7 had a history of cerebral infarction and was undergoing a recovery phase. Compared with DM patients with COVID-19 not treated in the ICU, there were more females in the ICU group (85.7% vs. 33.3%). More DM patients with COVID-19 admitted to the ICU (71.4%) had an exposure history of Wuhan than non-ICU patients (41.7%). There were more underlying disorders in the ICU group (100.0%) than the non-ICU group (58.3%). No obvious differences regarding mean age (63.0 years vs. 61.5 years) and the mean time of DM duration (5.1 years vs. 5.3 years) were observed in two groups. DM patients in the ICU group presented with more symptoms on admission, including fever (85.7%), cough (85.7%), and dyspnea (57.1%), but the average highest temperature in the two groups was similar (37.9°C vs. 37.9°C). The average time from illness onset to hospital admission was 7.6 days in the ICU group and 5.4 days in the non-ICU group. As the high proportion of female patients in the ICU group, we further divided 19 patients into the male group and female group to compare the baseline data (Table 2). Female patients showed older age (64.2 years vs. 59.7 years), more comorbidities (80.0% vs. 66.7%), and had more proteinuria (60.2% vs. 22.2%).

#### 3.2.2. Radiologic and Laboratory Findings

On admission, the mean level of glycated hemoglobin A1c (HbA1c) was higher in the ICU group than that in the non-ICU group (8.5% vs. 7.1%). There were more patients with proteinuria in the ICU group (57.1% vs. 33.3%). Two patients had elevated levels of creatines (patient 9 and patient 17). Lymphocytes were below the normal range in four ICU patients (57.1%) and two non-ICU patients (16.7%). The levels of blood urea nitrogen (BUN) were increased in three ICU patients (42.9%), and all non-ICU patients had normal BUN. Four ICU patients (57.1%) had D − dimer > 1 *μ*g/ml, while only two non-ICU patients (16.7%) had this abnormality. Two patients had elevated levels of creatine kinase (CK) (patient 1 and patient 3). Lactate dehydrogenase (LDH) was increased in four ICU patients (57.1%) and two non-ICU patients (16.7%). The details of laboratory results are shown in Table 2. More ICU patients (100%) showed bilateral pneumonia lesions than non-ICU patients (58.3%).

#### 3.2.3. Treatment and Outcomes

Antivirals were given to 18 patients (94.7%) as initiated therapy. Six DM patients admitted to the ICU (85.7%) received antibiotic therapy, while no non-ICU patients used antibiotics. Methylprednisolone sodium succinate was given to six ICU patients (85.7%) and one non-ICU patient who showed rapid progression. Six ICU patients (85.7%) received immunoglobulin therapy. Twelve patients (63.2%) changed diabetic therapy during hospitalization, and patients admitted to the ICU all used insulin (Table 3 and Supplementary material (available here)). No DM patients received support from an invasive mechanical ventilator. Four ICU patients (57.1%) had complications during hospitalization, including acute cardiac injury (14.3%), liver injury (28.6%), and allergic eruption (14.3%). As of March 14, all 19 patients have been discharged, and no death occurred. Notably, the average time from symptom onset to a negative PCR result was much longer in patients admitted to the ICU (30.6 days vs. 17.9 days).

## 4. Discussion

In the present study, we provided epidemiological and clinical data of 241 patients with COVID-19, including detailed information of 19 DM patients, in Changsha, Hunan Province, China. The average age was 62.1 years old in DM patients with COVID-19, which was older than non-DM patients. Compared with non-DM patients, DM patients had more comorbidities, particularly hypertension and CHD. DM patients had a higher incidence of proteinuria and higher level of CRP on admission. There were more patients in the DM group admitted to the ICU than the non-DM group. Moreover, female DM patients were vulnerable to severe COVID-19. Notably, DM patients had a longer time for viral clearance, but there was no difference regarding the mortality. As for details of DM patients with COVID-19, about half of the DM patients had an exposure history of Wuhan before illness onset. Hypertension was the most common underlying disease. Fever and cough were the most frequent symptoms on admission. The mean time of DM duration was 5.2 years. Three patients were diagnosed with DM for the first time.

In our study, the rate of severe COVID-19 (36.8%) in DM patients is relatively higher than non-DM patients and higher than that in the general population with rates of 16.0-32.0% [4–6]. The susceptibility of severe disease in DM patients may be attributed to several mechanisms. First, hyperglycemia could increase glucose concentrations in airway secretion and may lead to an elevated viral replication [7]. Second, DM patients usually present with suppressed immune response, such as the low levels of alexin C3 and alexin C4, which may reduce the capability of viral clearance and increase the risk of severe disease [8, 9]. Besides that, the altered glucose metabolism and oxidative stress could reduce the phagocytic activity of neutrophils and macrophages, impair T cell-mediated immunity, and decrease the secretion of cytokines, which impede the recognition and clearance of infected cells [10]. Moreover, a study indicated that SARS-CoV-2 could directly bind to ACE2 receptor of cells with high affinity [11], and the expression of ACE2 may be increased in DM patients treated with ACEI, ARB, and thiazolidinediones, which may be associated with increased risk of developing severe and fatal COVID-19 [12]. However, in our series, there were only two patients used ACEI or ARB before admission, and no patients used thiazolidinediones, and the relationship between ACE2 and severe COVID-19 in DM patients needs further clinical confirmation.

Notably, the proportion of female patients was much higher in the DM patients admitted to the ICU, which is different from the general population with COVID-19 as previous studies found more males in severe patients with COVID-19 [5, 6]. In our series, female DM patients were older, had more comorbidities, and had a longer duration of DM disease. In addition, more female patients showed proteinuria, which suggested diabetic nephropathy. All the above may be associated with severe COVID-19, as studies indicated older age and comorbidities are risk factors for poor outcomes of COVID-19 [4, 13]. A large systemic review indicated female patients with DM might be more vulnerable to CHD partly due to the low level of testosterone [14]. Besides that, female DM patients showed more comorbidities including nephropathy, CHD, stroke, depression, and anxiety than male DM patients, which may be associated with psychosocial factors, genetic predisposition, and sex hormones [15]. Whether similar factors influence the severity of COVID-19 in DM patients needs investigation, and the female preference in DM patients with severe COVID-19 still requires further confirmation because of the limited sample size in our study.

In terms of laboratory results on admission, DM patients in the ICU group had more lymphopenia, increased BUN, a D − dimer > 1 *μ*g/ml, higher levels of CRP, and elevated LDH compared with DM patients in the non-ICU group. Theses laboratory abnormalities between severe and nonsevere patients with DM were in accordance with features in general population with COVID-19, as studies showed severe COVID-19 patients usually presented with more decreased lymphocyte counts, increased inflammatory factors, elevated D-dimer, and higher levels of myocardial zymogram [16, 17]. Interestingly, DM patients admitted to the ICU had higher levels of HbA1c than non-ICU patients in our study, while the average DM duration was similar in two groups. Usually, HbA1c testing takes an important role in the management of DM, which represents the control of blood glucose over the past 2-3 months [18]. The higher levels of HbA1c in severe patients from our series indicated that poor glycemic control in DM patients could lead to a severe COVID-19, which also suggested that hyperglycemia may play a vital role in poor outcomes of DM patients with COVID-19. Moreover, in the current study, three patients were diagnosed with DM for the first time mainly based on their results of HbA1c, because hyperglycemia may be caused by stress, infection, and the use of corticosteroids [19–21]. Thus, we think it is beneficial to take a HbA1c test for DM patients with COVID-19 on admission or patients with abnormal blood glucose to assess the risk for severe disease. In addition to HbA1c, there were more DM patients with proteinuria in the ICU group, which indicates that the nephropathy may be associated with severe COVID-19. But we could not identify that the proteinuria in our case series were signs of DM nephropathy as most of these patients had hypertension meanwhile. In all, the examination of urinalysis is also important for DM patients with COVID-19.

Though the high proportion of severe patients in our series, no death occurred and all of them had been discharged. The good outcomes of these DM patients with COVID-19 may be attributed to timely combined interventions including the use of antivirals, antibiotics, corticosteroids, intravenous immunoglobulin, and oxygen support. At present, no drug has been proved to be specific for SARS-CoV-2 infection, and systemic therapy is recommended by the National Health Commission of China and WHO [22, 23]. Moreover, it is essential to monitor and control the blood glucose for DM patients with COVID-19. In our case series, more than half of the patients changed diabetic therapy during hospitalization, and all patients admitted to the ICU received insulin. The regulation of diabetic therapy was under the guidance of endocrinologists. According to the guidelines from Chinese Diabetes Society [24] and front-line physicians in China [25], the goals for blood glucose control are less stringent in COVID-19 patients admitted to the ICU: a fasting blood glucose of 7.8-10.0 mmol/l and a 2-hour postprandial of 7.8-13.9 mmol/l should be achieved. While notably, DM patients admitted to the ICU had much longer time from symptoms onset to a negative RT-PCR result of SARS-CoV-2, which indicates the viral clearance in severe COVID-19 patients with DM needs more time.

This study has several limitations. First, our study is single center and the number of DM patients was really limited. Involving multicenter or more patients may better investigate the clinical features of DM patients with COVID-19 and confirm the risk factors of severe disease. Second, most patients were not checked for diabetic complications, such as diabetic retinopathy, neuropathy, and angiopathy. Further studies may be needed to find out the relationship between diabetic complications and the severity of COVID-19.

## 5. Conclusions

In conclusion, DM patients with COVID-19 are vulnerable to severe disease, especially female patients. High levels of HbA1c and proteinuria could be potential risk factors for severe COVID-19 in DM patients. In addition to timely combined interventions including antivirals, antibiotics, corticosteroids, immunoglobulin, and oxygen support, the control of blood glucose and proper diabetic therapy is essential to improve the prognosis of severe COVID-19 patients with DM.

## Figures and Tables

**Table 1:** Comparisons between COVID-19 patients with DM and without DM.

	Total (*n* = 241)	DM (*n* = 19)	Non-DM (*n* = 222)	*P*
Age, years	45.2 (1-84)	62.1 (42-76)	43.8 (1-84)	<0.0001
Sex				
Male	122 (50.6%)	9 (47.4%)	113 (50.9%)	0.768
Female	119 (49.4%)	10 (52.6%)	109 (49.1%)	
Wuhan exposure	118 (49.0%)	10 (52.6%)	108 (48.6%)	0.739
Comorbidity	80 (33.2%)	14 (73.7%)	66 (29.7%)	<0.0001
Hypertension	36 (14.9%)	12 (63.2%)	24 (10.8%)	<0.0001
Coronary heart disease	9 (3.7%)	3 (15.8%)	6 (2.7%)	0.024
Chronic liver disease	11 (4.6%)	3 (15.8%)	8 (3.6%)	0.061
Cerebral infarction	7 (2.9%)	1 (5.3%)	6 (2.7%)	1.000
Admission symptoms				
Cough	132 (54.8%)	9 (47.4%)	123 (55.4%)	0.499
Dyspnea	27 (11.2%)	5 (26.3%)	22 (9.9%)	0.072
Fever^a^	169 (70.1%)	13(68.4%)	156 (70.3%)	0.866
Highest temperature, °C	37.9 (36.1-40.3)	37.9 (36.1-39.3)	37.9 (36.4-40.3)	0.946
Admission measures				
Proteinuria	21 (8.7%)	8 (42.1%)	13 (5.9%)	<0.0001
White blood cell count, ×10^9^/L	5.1 (0.8-19.0)	5.7 (0.8-12.0)	5.1 (1.8-19.0)	0.297
Neutrophil count, ×10^9^/L	3.4 (0.3-17.4)	4.2 (0.7-11.1)	3.3 (0.3-17.4)	0.109
Lymphocyte count, ×10^9^/L	1.3 (0.1-9.5)	1.1 (0.1-3.0)	1.4 (0.2-9.5)	0.210
Creatinine, *μ*mol/L	59.9 (7.8-492.2)	69.3 (34.3-255.7)	59.1 (7.8-492.2)	0.264
Blood urea nitrogen, mmol/l	4.9 (1.4-16.9)	5.8 (3.0-16.9)	4.8 (1.4-14.7)	0.225
ALT, U/L	22.8 (4.9-87.7)	23.4 (8.1-68.4)	22.7 (4.9-87.7)	0.824
AST, U/L	27.3 (5.5-82.0)	28.4 (14.5-52.6)	27.2 (5.5-82.0)	0.669
Creatine kinase, U/L	113.9 (9.3-1313.8)	108.3 (9.3-501.9)	114.4 (22.7-1313.8)	0.863
Lactate dehydrogenase, U/L	190.7 (82.5-539.9)	200.8 (120.7-319.4)	189.8 (82.5-539.9)	0.552
D-dimer, *μ*g/ml	0.8 (0.0-12.6)	1.1 (0.1-6.5)	0.79 (0.0-12.6)	0.496
C-creative protein, mg/L	27.3 (0.1-106.2)	44.8 (1.0-80.3)	25.8 (0.1-106.2)	0.003
Bilateral pneumonia	171 (71.0%)	14 (73.7%)	157 (70.7%)	0.785
ICU admission	42 (17.4%)	7 (36.8%)	35 (15.8%)	0.045
Time from symptoms onset to admission, days	6.2 (1-30)	6.4 (1-15)	6.2 (1-30)	0.867
Time from symptoms onset to negative RT-PCR result, days	15.9 (3-50)	22.6 (8-50)	14.3 (3-38)	0.004
Death	2 (0.8%)	0	2 (0.9%)	1.000

Data are presented as mean (range) or *n* (%). Abbreviations: DM: diabetes mellitus; ICU: intensive care unit; RT-PCR: reverse transcription-polymerase chain reaction; ALT: alanine aminotransferase; AST: aspartate aminotransferase. ^a^Defined as a axillary temperature of greater than 37.3°C.

**Table 2:** Clinical characteristics of DM patients with COVID-19.

	Classification based on severity		Classification based on gender		
	ICU (*n* = 7)	Non-ICU (*n* = 12)	Female (*n* = 10)	Male (*n* = 9)	Reference range
Age, years	63.0 (42-73)	61.5 (46-76)	64.2 (46-73)	59.7 (42-76)	
Sex					
Male	1 (14.3%)	8 (66.7%)	-	-	
Female	6 (85.7%)	4 (33.3%)	-	-	
Wuhan exposure	5 (71.4%)	5 (41.7%)	6 (60.0%)	4 (44.4%)	
Comorbidity	7 (100.0%)	7 (58.3%)	8 (80.0%)	6 (66.7%)	
Hypertension	5 (71.4%)	7 (58.3%)	7 (70.0%)	5 (55.6%)	
Coronary heart disease	1 (14.3%)	2 (16.7%)	2 (20.0%)	1 (11.1%)	
Chronic liver disease	3 (42.9%)	0	2 (20.0%)	1 (11.1%)	
Cerebral infarction	1 (14.3%)	0	1 (10.0%)	0	
Time of DM duration, year(s)	5.1 (0-15)	5.3 (0.5-10)	6.1 (0-15)	3.8 (0-10)	
Admission symptoms					
Cough	6 (85.7%)	3 (25.0%)	6 (60.0%)	3 (33.3%)	
Dyspnea	5 (71.4%)	0	4 (40.0%)	1 (11.1%)	
Fever^a^	6 (85.7%)	7 (58.3%)	9 (90.0%)	4 (44.4%)	
Highest temperature, °C	37.9 (36.4-39.0)	37.9 (36.1-39.3)	38.1 (37.1-39)	37.6 (36.1-39)	
Admission measures					
HbA1c, %	8.5 (7.5-9.5)	7.1 (6.2-7.5)	7.9 (7.0-9.5)	7.3 (6.2-9.5)	3-6.5
Proteinuria	4 (57.1%)	4 (33.3%)	6 (60.0%)	2 (22.2%)	
White blood cell count, ×10^9^/L	6.3 (0.8-12.0)	5.4 (2.6-9.9)	5.7 (0.8-12.0)	5.8 (4.1-9.9)	4-10
Neutrophil count, ×10^9^/L	4.8 (0.7-11.1)	3.8 (1.9-8.2)	4.5 (0.7-11.1)	4.9 (2.8-8.2)	2-7
Lymphocyte count, ×10^9^/L	1.1 (0.1-3.0)	1.1 (0.6-1.9)	0.9 (0.1-1.9)	1.4 (0.6-3.0)	0.8-4
Creatinine, *μ*mol/L	80.0 (40.3-255.7)	63.1 (34.3-124.1)	72.9 (34.3-255.7)	65.3 (42.8-124.1)	54-106
Blood urea nitrogen, mmol/l	7.6 (3.0-16.9)	4.7 (3.1-6.9)	6.7 (3.2-16.9)	4.7 (3.0-7.2)	2.86-8.20
ALT, U/L	20.0 (8.2-44.6)	25.3 (8.1-68.4)	21.6 (8.1-44.6)	25.4 (8.5-68.4)	0-42
AST, U/L	27.9 (14.5-52.6)	28.6 (15.5-43.5)	29.4 (14.5-52.6)	27.2 (15.5-43.5)	0-37
Creatine kinase, U/L	125.0 (9.3-501.9)	98.6 (44.8-193.5)	124.5 (9.3-501.9)	90.4 (44.8-193.5)	10-190
Lactate dehydrogenase, U/L	243.0 (136.4-319.4)	176.1 (120.7-244.7)	233.7 (133.7-319.4)	164.2 (120.7-273.9)	135-225
D-dimer, *μ*g/ml	1.9 (0.2-6.5)	0.6 (0.1-3.9)	1.7 (0.1-6.5)	0.4 (0.1-1.2)	0-1
C-creative protein, mg/L	52.0 (1.0-77.4)	40.6 (2.7-80.3)	55.7 (16.5-77.4)	32.6 (1.0-80.3)	0-8
Bilateral pneumonia	7 (100.0%)	7 (58.3%)	10 (100.0%)	4 (44.4%)	
Time from symptoms onset to admission, days	7.6 (1-12)	5.4 (1-15)	6.8 (3-12)	5.6 (1-15)	

Data are presented as mean (range) or *n* (%). Abbreviations: DM: diabetes mellitus; ICU: intensive care unit; RT-PCR: reverse transcription-polymerase chain reaction; ALT: alanine aminotransferase; AST: aspartate aminotransferase. ^a^Defined as a axillary temperature of greater than 37.3°C.

**Table 3:** Treatment and outcomes of DM patients with COVID-19.

	Total (*n* = 19)	ICU (*n* = 7)	Non-ICU (*n* = 12)
Complications	7 (36.8%)	4 (57.1%)	3 (25.0%)
Acute cardiac injury	1 (5.3%)	1 (14.3%)	0
Acute liver injury	5 (26.3%)	2 (28.6%)	3 (25.0%)
Allergic eruption	1 (5.3%)	1 (14.3%)	0
Treatment			
Antivirals	18 (94.7%)	7 (100.0%)	11 (91.7%)
Antibiotics	6 (31.6%)	6 (85.7%)	0
Corticosteroid	7 (36.8%)	6 (85.7%)	1 (8.3%)
Immunoglobulin	6 (31.6%)	6 (85.7%)	0
Insulin	8 (42.1%)	7 (100.0%)	1 (8.3%)
Oxygen support			
Nasal cannula	14 (73.7%)	4 (57.1%)	10 (83.3%)
High-flow nasal cannula	3 (15.85)	3 (42.9%)	0
Time from symptoms onset to negative RT-PCR result, days	22.6 (8-50)	30.6 (19-50)	17.9 (8-31)

Data are presented as mean (range) or *n* (%). Abbreviations: DM: diabetes mellitus; RT-PCR: reverse transcription-polymerase chain reaction.

## Data Availability

The clinical data of patients used to support the findings of this study are available from the corresponding author upon request.

## Supplementary Materials

Supplementary Materials See Table S1 in the Supplementary Material for comprehensive details of 19 DM patients with COVID-19.
