# Supplementary material for: Clinical Findings in Diabetes Mellitus Patients with COVID-19
Source: J Diabetes Res. 2021 Jan 8;2021:7830136. doi: 10.1155/2021/7830136 (PMC7811569; doi:10.1155/2021/7830136)
Supplement: Supplementary Materials — See Table S1 in the Supplementary Material for comprehensive details of 19 DM patients with COVID-19. [file 7830136.f1.docx]

**Table S1. Detailed information of DM patients with COVID-19.**

| **Patient No.** | **Sex** | **Age** | **Comorbidities** | | **Time of diabetes duration, years** | **Previous**  **diabetic therapy** | **Diabetic therapy in hospital** | **HbA1c level** | **Proteinuria** | **Symptoms on admission** | **CT findings** | **Clinical classification** ^b^ | **Treatment** | **Oxygen support** | **Symptom onset to admission, days** | **Symptom onset to negative RT-PCR result, days** |
| --- | --- | --- | --- | --- | --- | --- | --- | --- | --- | --- | --- | --- | --- | --- | --- | --- |
|  |  |  | **Hypertension** | **Coronary heart disease** |  |  |  |  |  |  |  |  |  |  |  |  |
| 1 | Male | 67 | Yes | No | 3 | Acarbose | Acarbose | 7 | **-** | Fever, cough | Focal consolidation | Moderate | Lopinavir & Ritonavir  Interferon atomization | Nasal cannula | 1 | 13 |
| 2 | Male | 59 | Yes | No | 5 | Metformin | Metformin | 6.8 | **-** | Fever | GGOs  Focal consolidation | Moderate | Lopinavir & Ritonavir  Interferon atomization | Nasal cannula | 15 | 19 |
| 3 | Female | 64 | Yes | No | 0^a^ | None | Insulin | 8.2 | **-** | Fever, cough, | GGOs  Focal consolidation | Critical | Lopinavir & Ritonavir  Interferon atomization  Moxifloxacin  Methylprednisolone sodium succinate  Immunoglobulin | High-flow nasal cannula | 6 | 21 |
| 4 | Female | 61 | Yes | No | 0.5 | Acarbose | Acarbose | 7 | **-** | Fever, | Nodular opacities | Moderate | Arbidol  Interferon atomization  Methylprednisolone sodium succinate | Nasal cannula | 3 | 19 |
| 5 | Female | 71 | Yes | No | 7 | Dapagliflozin  Glimepiride  Metformin | Dapagliflozin  Acarbose  Metformin | 7.4 | **+** | Fatigue | GGOs | Moderate | Arbidol  Interferon atomization | Nasal cannula | 4 | 12 |
| 6 | Male | 55 | Yes | No | Not clear | Miglitol  Metformin | Acarbose  Metformin | 7.5 | **-** | None | Nodular opacities | Moderate | Lopinavir & Ritonavir  Interferon atomization | Nasal cannula | N/A^c^ | 13^d^ |
| 7 | Female | 57 | Yes | No | 0^a^ | None | Insulin | 7.5 | **+** | Fever, diarrhea | GGOs  Nodular opacities | Severe | Lopinavir & Ritonavir  Interferon atomization  Moxifloxacin  Methylprednisolone sodium succinate  Immunoglobulin | Nasal cannula | 8 | 19 |
| 8 | Male | 48 | Yes | Yes | Not clear | Acarbose  Metformin | Acarbose  Metformin | 6.4 | **-** | Fever, cough | GGOs  Focal consolidation | Moderate | Lopinavir & Ritonavir  Interferon atomization | Nasal cannula | 7 | 17 |
| 9 | Male | 70 | Yes | No | 3 | Acarbose  Metformin | Acarbose  Metformin | 6.2 | **-** | None | GGOs | Moderate | Lopinavir & Ritonavir  Interferon atomization | Nasal cannula | N/A^c^ | 8^d^ |
| 10 | Female | 72 | Yes | No | 2 | Gliclazide | Insulin | 8.0 | **+** | Fever, cough, dyspnea | GGOs  Focal consolidation | Severe | Lopinavir & Ritonavir  Moxifloxacin  Methylprednisolone sodium succinate  Immunoglobulin | Nasal cannula | 12 | 20 |
| 11 | Female | 66 | Yes | Yes | 9 | Metformin  Gliclazide | Insulin Acarbose | 8.4 | **+** | Fever, cough, dyspnea | GGOs  Focal consolidation | Critical | Lopinavir & Ritonavir  Interferon atomization  Piperacillin-tazobactam  Linezolid  Methylprednisolone sodium succinate  Immunoglobulin | High-flow nasal cannula | 10 | 30 |
| 12 | Female | 65 | No | No | 7 | Acarbose | Acarbose | 7 | **-** | Fever,  Cough | GGOs  Focal consolidation | Moderate | Lopinavir & Ritonavir  Interferon atomization | Nasal cannula | 5 | 11 |
| 13 | Male | 76 | No | No | 10 | Acarbose  Gliclazide  Sitaglipin | Acarbose  Gliclazide  Sitaglipin | 7.5 | **+** | Fever | GGOs | Moderate | Interferon atomization | Nasal cannula | 4 | 29 |
| 14 | Female | 73 | No | Yes | 10 | Insulin | Increased Insulin dosage | 9.5 | **-** | Fever, cough, dyspnea | GGOs | Severe | Lopinavir & Ritonavir  Interferon atomization  Moxifloxacin  Methylprednisolone sodium succinate  Immunoglobulin | Nasal cannula | 8 | 38 |
| 15 | Female | 46 | No | No | 10 | Irregular | Gliquidone | 7.5 | **+** | Fever | Focal consolidation | Moderate | Lopinavir & Ritonavir  Interferon atomization | Nasal cannula | 4 | 31 |
| 16 | Male | 58 | No | No | 2 | Insulin  Metformin | Insulin  Metformin Acarbose | 7.2 | **+** | None | GGOs | Moderate | No-special | None | N/A^c^ | 26^d^ |
| 17 | Female | 67 | Yes | No | 15 | Metformin  Gliquidone | Metformin  Insulin | 8.2 | **+** | Fever, cough, dyspnea | GGOs  Pleural effusion | Severe | Lopinavir & Ritonavir  Interferon atomization  Piperacillin-tazobactam  Methylprednisolone sodium succinate  Immunoglobulin | High-flow nasal cannula | 8 | 36 |
| 18 | Male | 42 | No | No | 0^a^ | None | Metformin  Acarbose  Insulin | 9.5 | **-** | Cough, dyspnea | GGOs  Focal consolidation | Severe | Lopinavir & Ritonavir | Nasal cannula | 1 | 50 |
| 19 | Male | 62 | No | No | Not clear | Irregular | Acarbose  Insulin | 7.3 | **-** | None | Clear | Mild | Lopinavir & Ritonavir | None | N/A^c^ | 17^d^ |

Abbreviation: DM, diabetes mellitus; HbA1c, glycated hemoglobin A1c; CT, computerized tomography; GGO, Ground-glass opacities; RT-PCR, reverse transcription-polymerase chain reaction.

a Three patients were diagnosed with diabetes mellitus for the first time.

b The patients were classified into different clinical types based on the New Coronavirus Pneumonia Prevention and Control Program (Version six) from the National Health Commission of China, and severe and critical patients were admitted to the intensive care unit.

c N/A, not applicable, these patients had no symptoms before admission and they were screened because of a history of contact.

d The time of these patients were calculated from diagnosis to negative RT-PCR result, as they showed no symptoms before admission.
